# A Longitudinal Study of a Large Clinical Cohort of Patients with Lyme Disease and Tick-Borne Co-Infections Treated with Combination Antibiotics

**DOI:** 10.3390/microorganisms11092152

**Published:** 2023-08-24

**Authors:** David Xi, Abbie Thoma, Minha Rajput-Ray, Anne Madigan, Gordana Avramovic, Kunal Garg, Leona Gilbert, John S. Lambert

**Affiliations:** 1 School of Medicine, University College Dublin, D04 V1W8 Dublin, Ireland; davidxjy.97@gmail.com (D.X.); abbie.thoma@ucdconnect.ie (A.T.); annemadigan10@gmail.com (A.M.); gavramovic@mater.ie (G.A.); 2 Curaidh Clinic: Innovative Solutions for Pain, Chronic Disease and Work Health, Perthshire PH2 8EH, UK; drminha@curaidh.com; 3 Te?ted Oy, 40100 Jyväskylä, Finland; kunal.garg@tezted.com (K.G.); leona.gilbert@tezted.com (L.G.); 4 Infectious Diseases Department, Mater Misericordiae University Hospital, D07 R2WY Dublin, Ireland; 5 Infectious Diseases Department, The Rotunda Hospital, D01 P5W9 Dublin, Ireland

**Keywords:** Lyme disease, tick-borne infections, tick-borne co-infections, Lyme symptoms, Borrelia, Babesia, Bartonella, Ehrlichia, Rickettsia

## Abstract

The rising prevalence of tick-borne infections (TBIs) necessitates further attention. This study retrospectively investigated the types of TBIs, symptoms, and if combination antibiotics were helpful within a patient cohort at an infectious disease clinic in Ireland. In this chart audit of 301 individuals (184 female, 117 male) tested for TBIs, 140 (46.51%) had positive antibody responses for TBIs from an ELISA (enzyme-linked immunoassay) that was based on a modified two-tiered testing protocol. A total of 93 (66.43%) patients had positive antibody responses to one TBI: 83 (59.29%) for Borrelia, 7 (5.00%) for Rickettsia, and 1 (0.71%) each for either Babesia, Bartonella, or Ehrlichia. The remaining 47 (33.57%) patients were infected with multiple TBIs. These patients were treated with combination antibiotics and monitored at two subsequent follow-ups. Only 2 of 101 patients (1.98%) had discontinued treatment by the second follow-up. In the first follow-up with 118 patients, 70 (59.32%) reported pain and 48 (40.68%) had neurological symptoms. In the next follow-up of 101 patients, 41 (40.59%) had pain while 30 (29.70%) had neurological symptoms. There were statistically significant reductions in the incidence of pain (41.43%) and neurological (37.50%) symptoms between follow-ups. Thus, our study demonstrates that combination antibiotics effectively relieve TBI symptoms with good patient tolerance.

## 1. Introduction

Globally, tick-borne infections (TBIs) are increasingly recognized as an important cause of zoonotic diseases [1]. In fact, tick-derived pathogens contribute to the bulk of vector-borne infections in Europe, Asia, and the temperate regions of North America [2]. Global warming is a significant driver of tick population growth, enabling their migration to higher altitudes and latitudes [3,4,5]. With the exception of Lyme borreliosis, tick-borne diseases are sometimes overlooked among vector-borne diseases [1]. Complex vector–pathogen–host interactions make an estimation of the national incidence challenging [5,6,7]. Tick-borne diseases can be concentrated in rural or agricultural settings [5,7], which might not receive adequate public health attention [1]. With a broad spectrum of microorganisms within ticks [6,7], further research into TBIs is crucial to improve diagnosis, treatment, and eradication.

The focus of research has mainly centered around the *Borrelia burgdorferi sensu lato* complex, which causes Lyme disease; the most prevalent tick-borne disease world-wide [3,4]. Within the *B. burgdorferi sensu lato* complex, the main pathogenic species are *B. burgdorferi sensu stricto* and *B. mayonii* in North America, and *B. afzelii* and *B. garinii* in Europe and Asia [8]. More recent surveillance data from the US estimated that 476,000 patients were treated for Lyme disease annually from 2010 to 2018 [9]. Within Europe, a systematic review by Vandekerckhove and colleagues [10] discovered a rising trend in the national incidence of Lyme disease in Norway and Finland. In another study, three countries, Switzerland, Belgium, and the Netherlands, recorded a national incidence of more than 100 per 100,000 population per year [7]. Limited data are available from countries such as Ireland, Portugal, and Spain [7,10]. A retrospective cohort study by Forde and co-workers [11] from 2012 to 2016 estimated the incidence to be 1.15 per 100,000 population per year for those between the ages of 2 and 18 in Ireland. In Ireland, the national incidence of tick-borne illnesses, such as Lyme disease, can be hard to estimate, as only Lyme Neuroborreliosis is a notifiable disease [12]. There were four notifications in 2021 in Ireland and the national neuroborreliosis notification rate is 0.08 per 100,000 population [12].

Lyme disease symptoms can be categorized as early localized, early disseminated, or late disseminated [13]. Initial symptoms of Lyme borreliosis usually appear 2–30 days after a tick bite [5]. Constitutional symptoms such as fever, malaise, muscle and joint aches, and *erythema migrans* rash, are described in the early stages of the disease [2,4,7,8,13]. An *erythema migrans* rash is a circular or ovoid erythematous lesion with a central clearing that resembles a target sign. It develops on average 7 days after a tick bite, but studies have reported the appearance of *erythema migrans* from 1 to up to 36 days after a Borrelia infection [8,14]. While *erythema migrans* is a classic sign of Lyme disease, it is not seen in all patients [4,7,8]. The early-disseminated stage usually begins within days to weeks and can manifest as multiple *erythema migrans*, Lyme carditis, or neurological deficits [4,7,8,13]. Bannwarth syndrome, a meningoradiculoneuritis due to Lyme neuroborreliosis, is one of the most common disease manifestations after *erythema migrans* [8,15,16]. Lyme carditis can lead to complications like atrioventricular blocks, including third-degree heart blocks, which can be fatal if untreated [8,17]. Lyme arthritis is among the most common late symptoms of Lyme disease [8]. Another late manifestation of Lyme borreliosis is *acrodermatitis chronica atrophicans*, a bluish-red dermatological discoloration of the extremities that can lead to tissue atrophy if untreated [18].

The diagnosis of Lyme disease is aided by clinical manifestations, such as *erythema migrans* and a positive patient history of exposure to tick-endemic areas or tick bites [8]. Careful evaluation is advised, as several publications have found that only about 14–32% of patients in the US recalled receiving a tick bite, and some patients do not present with *erythema migrans* [4,7,8,14]. Serological testing with a standard or modified two-tier testing protocol can support a diagnosis. Standard two-tier testing involves an initial enzyme immunoassay and the subsequent utilization of Western blotting [8]. In the modified two-tier testing protocol, two enzyme immunoassays are used [8]. Both immunoassays need to be positive to support the diagnosis of Lyme disease [8]. The modified two-tier testing protocol is more sensitive at detecting early infections and less labor-intensive [8].

Ticks can concurrently carry other Borrelia subspecies or microbes in addition to *B. burgdorferi* [19,20,21,22,23]. Rickettsiosis, Ehrlichiosis, Babesiosis, and Bartonellosis are other notable TBIs [2,6]. An important consideration is that infections with these pathogens give rise to vague and non-specific symptoms, unlike *erythema migrans* with Lyme disease [6]. Clinical presentations cannot reliably distinguish co-infections from mono-infections or uninfected patients [24]. In the eastern United States, the majority of tick-borne co-infections are Lyme disease and human babesiosis, which can have confounding impacts on the disease course and severity [6,14,21,23]. Co-infections with both *B. burgdorferi* and *B. microti* can increase the duration and severity of Lyme disease in the early phase of illness [6]. *B. burgdorferi* and *B. microti* also have a synergistic relationship that causes the higher parasitemia of *B. microti* in mice [6]. Another study in Switzerland found co-infections of *B. burgdorferi* with the spotted fever group Rickettsiae [25]. These patients are more likely to present with non-specific symptoms, such as myalgia and fatigue. The authors recommended co-infections to be ruled out during diagnosis, especially in endemic areas [25].

The antibiotic treatment for Lyme disease is determined by multiple factors, such as age, antibiotic tolerance and hypersensitivity, the type of symptoms, and the presence of co-infections [8,26,27]. Doxycycline, amoxicillin, or cefuroxime are all recommended for the first-line treatment of Lyme disease [26,27]. However, using combination antibiotics to treat long-term Lyme disease symptoms is controversial [16,28]. Debilitating chronic symptoms, such as pain, fatigue, and neurological symptoms, can arise from a Lyme borreliosis infection [26,29,30]. One possible cause is persistent *B. burgdorferi* infection, as the bacteria possess immune-evasion mechanisms, such as hindering complement activation and phagocytosis [29], existing as metabolically inactive forms like round bodies, and bacterial biofilm creation [31]. Prolonged inflammation, autoimmunity, or permanent physiological damage from an infection are other proposed mechanisms for chronic symptoms [26,30]. Post-treatment Lyme disease syndrome has been used to describe the chronic symptoms that persist even with antibiotic treatment, and without clinical or laboratory evidence of infection [26,28]. The most widely debated hypothesis is “Chronic Lyme disease (CLD)”, which shares many similarities with post-treatment Lyme disease syndrome. There are two categories proposed for CLD: untreated CLD (CLD-U) and previously treated (CLD-PT), where the latter demands that CLD symptoms remain present continuously or in a relapsing/remitting pattern for a period of six months or more after therapy [32]. To date, there is no consensus on the suitability and duration of antibiotic treatment for the chronic symptoms of Lyme disease [16,28].

Given the increasing global prevalence of tick-borne illnesses, further research could help improve the management of infections and co-infections. Currently, there is a lack of updated research on the incidence of different TBIs within Ireland. In this study, we aimed to investigate the types of TBIs and symptoms within a cohort of 301 patients from an Irish infectious disease clinic. We categorized the types of single and multiple tick-borne infections faced in this cohort. Secondly, we investigated the efficacy and safety of using prolonged combination antibiotics for relieving chronic symptoms in this cohort. We focused on the most common symptoms faced by this patient cohort: muscle and joint pain, fatigue, and neurological symptoms.

## 2. Materials and Methods

### 2.1. Study Objectives

The aims of this study are:

1. To investigate the types of TBIs within a patient cohort at an infectious disease outpatient clinic in Ireland.
2. To evaluate the efficacy and safety of using prolonged combination antibiotics for resolving chronic symptoms from TBIs in this patient cohort.

### 2.2. Patient Recruitment

All the patients who presented to an infectious disease outpatient clinic at The Mater Misericordiae Hospital, Eccles Street, Dublin 7, Ireland, who were to be evaluated for Lyme disease and co-infections, were offered participation in this study, following the inclusion and exclusion criteria. These patients exhibited “Lyme-like” symptoms, non-specific flu-like illness with clinical suspicion of tick-borne infections [33]. For instance, patients might recollect tick bites, have been exposed to tick-endemic areas, or have developed a bull’s-eye rash. Below are the inclusion and exclusion criteria for the study.


**Inclusion Criteria**

**Exclusion Criteria**

Male and female patients, >16 years of age, with a documented positive clinical history of a “Lyme-like” illness [33].Be willing and able to provide written in-formed consent before study participation.Be willing and able to comply with the study protocol.Patients who have valid contact details.

Patients unable or unwilling to provide consent.


### 2.3. Serology Analysis

An ELISA platform was used to assess IgM and IgG antibody responses to Borrelia spp (*B. afzelii* and *B. garinii*), Borrelia persister forms, Babesia, Bartonella, Ehrlichia, and Rickettsia in this patient cohort using a modified two-tiered testing protocol. Serological testing was conducted using the TICKPLEX^®^ test at ArminLabs GmbH in Augsburg, Germany. TICKPLEX^®^ has the capability to assess IgM and IgG immune responses present in human serum samples against various species of *Borrelia burgdorferi sensu lato* in both spirochete and persistent forms, as well as against co-infections and opportunistic microbes. Specifically, TICKPLEX^®^ encompasses *Borrelia burgdorferi sensu stricto, Borrelia afzelii*, and *Borrelia garinii* in their spirochete and persistent forms. It also covers other pathogens, like *Babesia microti, Bartonella henselae, Ehrlichia chaffeensis, Rickettsia akari*, Coxsackievirus, Epstein–Barr virus, Human parvovirus B19, *Mycoplasma fermentans,* and *Mycoplasma pneumoniae* [34]. The serological results were compiled and entered into an Excel spreadsheet for the handling of the data and analysis. We indicated if patients had positive, weakly positive, or negative antibody responses to the microorganisms. Using the serological data, we categorized patients into those with one TBI and those with multiple TBIs.

### 2.4. Patient Symptom Monitoring

During the initial visit (T0) to this infectious disease clinic, a patient history, clinical examination, and the necessary clinical investigations were conducted. Combination antibiotic treatments were given after the clinical consultation. Although our protocol scheduled follow-ups at 3 and 6 months, some appointments were rescheduled due to the COVID-19 pandemic.

A first assessment questionnaire (Appendix A) with 56 questions was administered to all 301 patients. The first 14 questions covered personal information, tick bites, and consultations before arrival at the clinic. Questions 15 to 47 were related to patient symptoms and were split into these 6 categories: skin, general well-being, cardiac, rheumatological, neurological, and psychological. As there is no validated symptom-monitoring questionnaire for TBIs and co-infections, the questions for each category were prepared using the current knowledge of common Lyme disease clinical manifestations [4,7,8,13,15,16]. Patients were also asked to rate their general state of health on a scale of 1 to 10, where a higher score signified better health. The remaining questions were based on blood tests and treatment to date, and included a free-response question allowing patients to list any further symptoms not covered in the questionnaire. The responses to this questionnaire served (T0) as the baseline for symptom monitoring.

Patients who returned to the clinic at two subsequent follow-up time points (T1 and T2) were given a follow-up visit questionnaire with 15 questions (Appendix B). In this part, we focused on the patient-reported perception of their general state of health and the incidence of the three most common symptoms in this cohort at T1 and T2. Again, respondents were asked to rate their well-being from 1 (poorly) to 10 (feeling very good). They were also asked to list their most distressing symptoms.

### 2.5. Statistical Analysis of Symptom Severity

The results from the questionnaires were compiled and entered into an Excel spreadsheet for the handling of data. We utilized Python libraries, including SciPy [35], NumPy [36], Pandas [37], Matplotlib [38], and Seaborn [39], to analyze and visualize symptom ratings at the T0, T1, and T2 time points. Pandas was used to organize and preprocess the symptom-rating data. NumPy allowed us to perform calculations and transformations on the data. Matplotlib was used to create visualizations, such as line plots and bar graphs. Seaborn provided specialized plots, like boxplots, to better understand the distribution and variability of the symptom ratings.

We employed a two-sample Kolmogorov–Smirnov (K-S) and Mann–Whitney U tests for the statistical analysis. The two-sample Kolmogorov–Smirnov test assessed the dissimilarity between two distributions, with the K-S statistic ranging from 0 to 1 [40]. A higher K-S statistic indicated a greater dissimilarity between the distributions. In addition to the Kolmogorov–Smirnov (K-S), we used Mann–Whitney U tests with a paired *t*-test and Cohen’s *d* effect size to analyze the difference in symptom incidence between time points T1 and T2 [41,42,43,44,45,46].

The significance of the K-S, Mann–Whitney U, and paired *t*-test results were determined by evaluating the *p*-values. A *p*-value less than 0.05, 0.01, or 0.001 was considered significant, depending on the predefined significance level. A smaller *p*-value indicated stronger evidence against the null hypothesis, and suggested a significant difference between the distributions of symptom ratings at the different time points. Cohen’s *d* effect size quantified the standardized difference between the means, and provided insights into the magnitude of the differences. Effect sizes of *d* ≥ 0.2, *d* ≥ 0.5, *d* ≥ 0.8, and *d* ≥ 1 were considered small, medium, large, and very large, respectively. This allowed us to evaluate the practical significance or strength of the observed differences between T1 and T2.

### 2.6. Patients’ Antibiotic Tolerance

To assess the treatment response and tolerance, questions on antibiotic tolerance were asked and recorded in the follow-up questionnaires. Patients either continued with the antibiotics prescribed at T0, changed antibiotics, or discontinued antibiotics. These responses were labeled as A, B, and C, respectively, in the Excel spreadsheet that can be found at the link in the Supplementary Materials, Table S1. Clinical and laboratory tests such as renal and liver function tests were conducted at the initial visit and two follow-up visits to help assess patient tolerance. This information was entered into the same Excel spreadsheet (Supplementary Materials, Table S1). As the prolonged use of combination antibiotics can cause gut microbiome dysregulation, probiotics like kefir were also provided to the patients to help mitigate this.

### 2.7. Ethics Approval

This study received ethics approval from the Institutional Review Board of the Mater Misericordiae University Hospital (Institutional Review Board Reference: 1/378/1946). It complies with the study protocol (version 6), the EU CT Directive 2001/20/EC, GCP Commission Directive 2005/28/EC, ICH/GCP, the Declaration of Helsinki (1996 Version), and all other local and international applicable regulatory requirements.

## 3. Results

### 3.1. Patient Characteristics

A total of 301 patients, 184 (61.13%) females and 117 (38.87%) males, from ages 16 to 89 years old, presented to the infectious disease clinic over 15 months, from December 2019 to February 2022. Of the 301 patients who came to the clinic at T0, 227 (75.42%) resided in Ireland. Dublin was listed as the county of residence within Ireland for the highest number of patients (52 patients, 17.28%). For the other patients, they were from various counties, such as Kerry, Meath, Wexford, and Wicklow (Table 1). The remaining patient (0.33%) was from Aran Island. For the patients who resided outside of the Republic of Ireland, 68 (22.59%) came from the United Kingdom, 2 (0.66%) came from the United States, 1 (0.33%) came from Hungary, 1 (0.33%) came from New Zealand, and the remaining patient (0.33%) came from Germany (Table 1). One (0.33%) patient did not indicate their country of residence.

### 3.2. Patient Cohort’s Serology Results

Out of 301 patients, 140 patients (46.51%) were antibody-positive to TBI (Table 2), of which 93 (66.43%) were positive to one type of TBI. Of the positive cases, 83 individuals (59.29%) were solely infected with Borrelia, 7 individuals (5.00%) were antibody-positive for Rickettsia alone, and 3 individuals were infected solely with Babesia, Bartonella, or Ehrlichia (0.71% each) (Table 2). The remaining 47 patients (33.57%) were infected with multiple TBIs.

A total of 42 individuals (30.00%) had antibodies to Borrelia and co-infections with Babesia, Bartonella, Ehrlichia, or Rickettsia (Table 3). There were two (1.43%) individuals with Babesia and Rickettsia co-infections, and two were infected with either Bartonella and Rickettsia (0.71%), or Bartonella and Babesia (0.71%). One patient (0.71%) was antibody-positive for Babesia, Rickettsia, and Ehrlichia.

### 3.3. Tick Bites and Erythema Migrans

From the questionnaire, 73 (52.14%) patients who were antibody-positive recalled receiving a tick bite. Additionally, 65 patients (46.43%) did not experience a bull’s-eye rash, 40 (28.57%) confirmed developing a rash, and 31 (22.14%) were unsure.

### 3.4. Analysis of Symptom Severity at T0, T1, and T2

Of the 140 antibody-positive patients, 118 returned at the T1 follow-up and 101 returned at the T2 follow-up. The patients who did not return for the follow-ups either experienced symptom resolution or missed their appointments.

**Figure 1:**
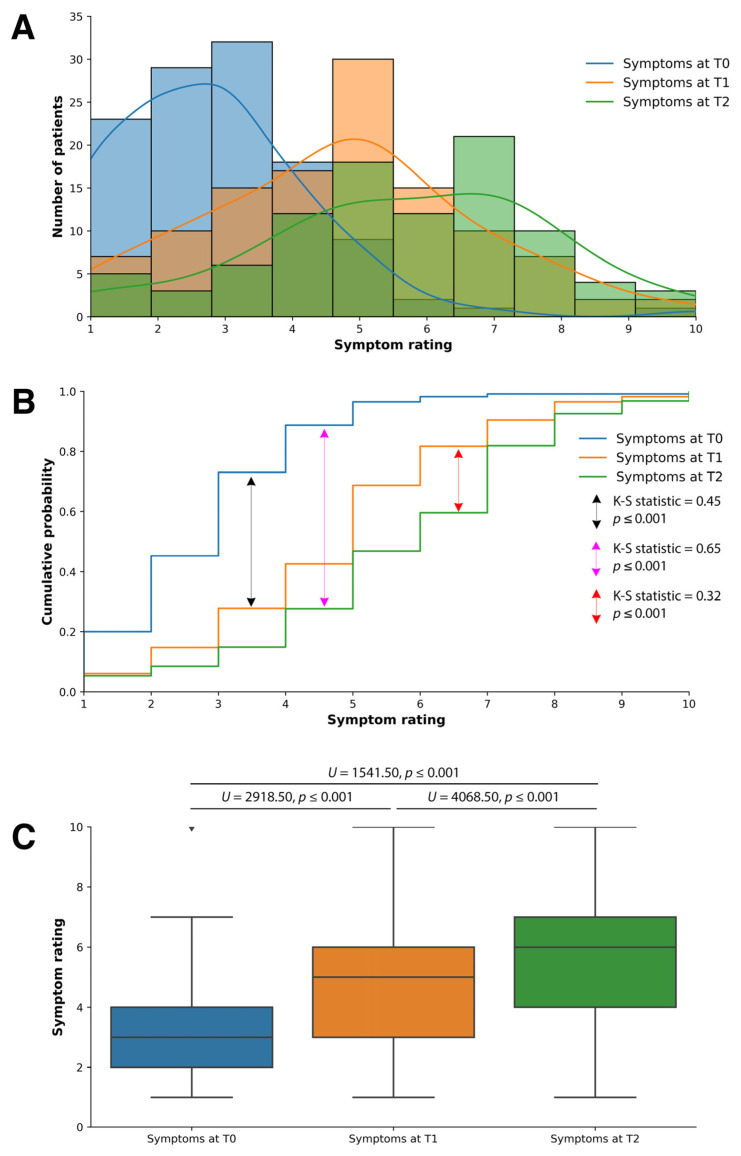
The patients’ overall health saw significant statistical changes from T0 to T2. (**A**) A histogram with kernel density estimation depicting the distribution of symptom ratings at time points T0, T1, and T2. (**B**) The cumulative probability distribution of the overall distribution and relative probabilities of the observed symptom ratings at the three time points. (**C**) A boxplot illustrates the median symptom-ratings increase from T0 to T1 to T2.

The patients exhibited significant improvements in their health status during the follow-up visits at time points T1 and T2, compared to the baseline measurement at time point T0 (Figure 1). Three graphical representations were employed to comprehensively understand the symptom-rating distribution (Figure 1). These graphical and statistical analyses collectively reinforce the evidence of significant health improvements observed in patients throughout their follow-up visits.

Firstly, Figure 1A illustrates a histogram with kernel density estimation depicting the distribution of symptom ratings at time points T0, T1, and T2. Using a scale from 1 to 10, where 1 is feeling very low or poorly and 10 is feeling very good, we saw a right shift of the symptom-rating distribution curve at T1 and T2 as compared to T0. There was a further shift to the right of the distribution curve from T1 to T2. At T0, the distribution curve peaked around scores 2–3, while the T1 distribution curve peaked at 5. The distribution curve for T2 has a plateau around scores 5–6, with the highest peak at score 7.

Secondly, Figure 1B shows the cumulative probability distribution, which offers insights into the overall distribution and relative probabilities of the observed symptom ratings at the three time points. To quantitatively assess the dissimilarity between the distributions at the three time points, a two-sample Kolmogorov–Smirnov (K-S) test was conducted, and the resulting *p*-value of 0.001 was used to determine the significance of this dissimilarity. There were statistically significant differences in the distribution of symptom ratings from T0 to T2 (K-S statistic = 0.65, *p* ≤ 0.001), T0 to T1 (K-S statistic = 0.45, *p* ≤ 0.001), and T1 to T2 (K-S statistic = 0.32, *p* ≤ 0.001). There was a greater difference in the distribution of symptom ratings from T0 to T1 than from T1 to T2.

Lastly, a boxplot (Figure 1C) is utilized to illustrate the increase in median symptom ratings from T0 to T2, T0 to T1, and T0 to T2. The median symptom ratings were approximately 3, 5, and 6 for T0, T1, and T2, respectively. A Mann–Whitney U test assessed the differences between the T0, T1, and T2 time points. There were statistically significant improvements in the median symptom ratings from T0 to T1 (U = 2918.50, *p* ≤ 0.001) and T2 (U = 1541.00, *p* ≤ 0.001) and from T1 to T2 (U = 4068.50, *p* ≤ 0.001).

### 3.5. Analysis of Chronic Persisting Symptoms

From the analysis of the questionnaire results, the three most common symptoms reported by the patients were pain, fatigue, and neurological symptoms, such as a tingling sensation in the limbs and memory defects. At the first follow-up at T1, out of 118 patients, 70 (59.32%) patients experienced pain, 48 (40.68%) reported neurological symptoms, and 57 (48.31%) had fatigue (Table 4).

Some patients’ symptoms had improved by the second follow-up (T2). Of the 101 patients who returned to the clinic at both T1 and T2, 41 (40.59%) patients were still suffering from pain, while neurological symptoms persisted in 30 (29.70%) patients, and 47 (46.53%) patients reported fatigue (Table 4).

The number of patients suffering from pain, neurological symptoms, and fatigue decreased by 41.43%, 37.50%, and 17.54%, respectively (Table 4). A significant statistical difference in pain and neurological symptoms between T1 and T2 was noticed, with a medium Cohen’s *d* effect size (Table 4). For fatigue, the difference in incidence between T1 and T2 was not statistically significant, and a small Cohen’s *d* effect size was observed (Table 4).

### 3.6. Antibiotic Treatment and Tolerance in Antibody-Positive Patients at T2

Among the 101 antibody-positive patients who returned for both T1 and T2 follow-ups, 95 (94.06%) patients were given a triple antibiotic combination regimen to be taken twice daily (Table 5). Most, 76 (72.65%) patients out of 101, were treated with a triple antibiotic regimen of 500 mg cefuroxime, 300 mg rifampicin, and 300 mg lymecycline. A total of six (5.94%) patients were given two antibiotic combination regimens (Table 5). A table summary of the number of patients prescribed with each type of combination antibiotic regimen is shown below (Table 5).

The duration of antibiotic treatment from T0 to T2 ranged from 12 weeks to 40 weeks. From the questionnaire responses, 77 of the 101 patients who returned for both follow-ups (76.24%) indicated that they still tolerated the antibiotic treatment (Supplementary Materials, Table S1). Due to side effects, 12 (11.88%) patients required a change in the antibiotic combination and 2 (1.98%) discontinued treatment. The remaining 10 patients did not provide an answer about antibiotic tolerance in the questionnaire at T2. Complete information on antibiotic duration, antibiotic tolerance, renal function tests, and liver function tests can be found in the supplementary materials (Supplementary Materials, Table S1).

One participant stopped the antibiotic treatment at 32 weeks but did not indicate the reason for discontinuation in the questionnaire. When asked at the first follow-up, she had previously tolerated the antibiotic treatment at 8 weeks. Her second renal and liver profile investigations showed that her CO2 total (31 mmol/L) and bilirubin (4 μmol) were outside the reference range of 22–29 mmol/L and 5–24 μmol, respectively (Supplementary Materials, Table S1). Her third liver profile showed an AST value of 17 I.U./L, which was lower than the reference range of 19–42 I.U./L. In isolation, these findings have an unclear clinical significance.

The last patient who ceased antibiotic treatment stopped at 16 weeks. She answered in her questionnaire that it was due to severe pain under her ribs that required a morphine injection. She restarted the antibiotic regimen at a lower dose 10 days later. She tolerated the antibiotic regimen at her first follow-up at 8 weeks. Her renal and liver function tests performed at 19 weeks did not show a significant deviation from her baseline at the initial visit, although her CO2 total was higher than the reference values, at 32 mmol/L (Supplementary Materials, Table S1).

## 4. Discussion

Our findings (Table 2 and Table 3) support the notion that infection from the *Borrelia burgdorferi* species is the most predominant TBI in Ireland, with most of the antibody-positive cases (59.29%) in this cohort being solely infected with Borrelia. A total of 42 out of 140 patients (30.00%) had co-infections of Borrelia with other TBIs, such as Babesia, Bartonella, Ehrlichia, and Rickettsia. This is notable, as earlier publications have established that co-infections with Ehrlichiosis and Babesiosis can complicate the disease course and treatment [6,14,21,23]. Furthermore, past research has also noted that *B. burgdorferi* can cause immune dysfunction and hinder the development of IgG-producing plasma cells [47]. One study also demonstrated that *B. burgdorferi* has immunosuppressive effects, as mice who were infected had less capability to produce antibodies against influenza [47]. Immune system derangements in TBIs could also impact the pathogenesis of tick-borne co-infections, as seen in the synergistic relationship between *B. burgdorferi* and *B. microti* co-infections, which cause higher serum levels of *B. microti* in mice [6]. It is important to consider and test for co-infections, especially in endemic areas and for those with unusual non-specific symptoms, or abnormal investigation results [25,27].

From our first assessment questionnaire results, only 52.14% of all the antibody-positive patients recalled receiving a tick bite. Some publications from the US found that only about 14–32% of patients recalled a tick bite [14]. Additionally, our questionnaire showed that 46.43% of the patients did not experience a bull’s-eye rash, and 22.14% were unsure if they had developed a rash. Only 28.57% of all the antibody-positive patients could confirm they had a rash. From the past literature, *erythema migrans* are not seen in all patients [4,7,8]. Our study thus highlights the importance of not relying solely on a positive tick bite or positive *erythema migrans* to consider a Lyme disease diagnosis.

Based on this patient cohort, we believe there is merit in using prolonged combination antibiotics to relieve the lingering symptoms from TBIs. For this patient cohort, the three most commonly reported patient symptoms were pain, fatigue, and neurological symptoms, such as a tingling sensation in the limbs and memory defects. These three symptoms were among the most reported persisting symptoms by others [26,30]. A total of 94.06% of the patients who returned to the clinic at both T1 and T2 had been prescribed three antibiotics, and the remaining 5.94% were given two antibiotics from T0 to T2. Although current guidelines by the IDSA (Infectious Diseases Society of America) and ILADS (International Lyme and Associated Diseases Society) sometimes differ on the optimum duration of antibiotic treatment, both do not recommend treatment beyond 6 weeks without clinical reassessment [27,48]. These guidelines also recommended single antibiotic treatment for Lyme disease in most circumstances [27,48]. Earlier studies were inconclusive for determining the efficacy of long-term combination antibiotics [16,28]. However, our study illustrated that treatment with prolonged combination antibiotics is effective and has a good safety profile (Supplementary Materials, Table S1). From the results of our questionnaire, many patients had a general improvement in symptom severity from T0 to T1 and subsequently from T1 to T2. We also demonstrate a statistically significant difference in the incidence of pain and neurological symptoms between T1 and T2. Most antibody-positive patients who returned for both follow-ups tolerated the prolonged use of combination antibiotics, and only two (1.98%) discontinued the antibiotic treatment. Other publications have also found combination antibiotics effective in clearing persister forms of *B. burgdorferi* [49]. Current guidelines should consider prolonged combination antibiotics as a treatment for Lyme disease and co-infections.

This study was a retrospective analysis of patients presenting in a clinical setting who were prescribed, on a case-by-case basis, an antibiotic regimen. With close monitoring, the individuals were assessed with regards to antibiotic tolerability, allergies, safety, and potential efficacy. As this was a preliminary study to highlight treatment safety and the improvement in patient well-being and symptoms, further research should be conducted to find the most effective combination antibiotic regimen for the various clinical manifestations of Lyme disease.

We discovered no statistically significant difference in the incidence of fatigue between T1 and T2. An earlier randomized controlled trial of 55 patients with severe fatigue 6 months after antibiotic treatment for Lyme disease by Krupp and colleagues [50] showed that IV ceftriaxone for 28 days improved symptoms. In our study, we used the incidence of fatigue, instead of assessing the reduction in fatigue severity, with a 11-item questionnaire like Krupp and colleagues [50]. Another difference is the route of administration, as patients were managed in an outpatient setting and were not given IV antibiotics. This could mean that fatigue is a chronic symptom of Lyme disease that requires specific management and a more sensitive assessment tool to monitor treatment effect.

A limitation of this study is the lack of validated patient-reported symptom questionnaire specific for Lyme disease or other tick-borne infections. Our questionnaires were created based on the existing research literature on the common clinical manifestations of Lyme disease and the clinical experience of specialists in this area. Using the questionnaires to monitor patient-reported symptoms, our study assessed the most important clinical symptoms in our patient cohort.

## 5. Conclusions

Our study established that most patients in this cohort were infected with the *Borrelia burgdorferi* species, and about a third had co-infections with other tick-borne pathogens. Approximately half of the patients recalled receiving a tick bite and developing a bull’s-eye rash. Pain, fatigue, and neurological symptoms were among the most common persistent symptoms faced by this cohort from the initial visit to T2. With the use of long-term combination antibiotics, we noted symptom resolution from the initial visit to T2 with good patient tolerance.

## Figures and Tables

**Table 1:** Patient characteristics.

Patient Characteristics	Number of Patients, *n* (Percentage)
**Gender**	
Male	117 (38.87)
Female	184 (61.13)
**Place of Residence in Ireland**	227 (75.42)
Aran Island	1 (0.33)
Carlow	3 (1.00)
Cavan	4 (1.33)
Clare	13 (4.32)
Cork	13 (4.32)
Donegal	13 (4.32)
Dublin	52 (17.28)
Fermanagh	3 (1.00)
Galway	10 (3.32)
Kerry	15 (4.98)
Kildare	1 (0.33)
Kilkenny	3 (1.00)
Laois	7 (2.33)
Leitrim	5 (1.66)
Limerick	4 (1.33)
Longford	4 (1.33)
Louth	7 (2.33)
Mayo	7 (2.33)
Meath	11 (3.65)
Monaghan	2 (0.66)
Offaly	3 (1.00)
Roscommon	3 (1.00)
Sligo	5 (1.66)
Tipperary	4 (1.33)
Waterford	6 (1.99)
Westmeath	6 (1.99)
Wexford	12 (3.99)
Wicklow	10 (3.32)
**Place of Residence Outside Ireland**	73 (24.25)
United Kingdom	68 (22.59)
United States	2 (0.66)
Hungary	1 (0.33)
Germany	1 (0.33)
New Zealand	1 (0.33)

**Table 2:** The number of antibody-positive patients with single TBI.

Types of TBIs	Number of Antibody-Positive Patients, *n* (Percentage)
Borrelia	83 (59.29)
Rickettsia	7 (5.00)
Babesia	1 (0.71)
Bartonella	1 (0.71)
Ehrlichia	1 (0.71)

**Table 3:** The number of antibody-positive patients with multiple TBIs.

Types of TBIs	Number of Antibody-Positive Patients, *n* (Percentage)
Borrelia combined with Babesia, Bartonella, Ehrlichia, or Rickettsia	42 (30.00)
Babesia and Rickettsia	2 (1.43)
Babesia, Rickettsia, and Ehrlichia	1 (0.71)
Bartonella and Rickettsia	1 (0.71)
Bartonella and Babesia	1 (0.71)

**Table 4:** Analysis of the incidence of pain, fatigue, and neurological symptoms between T1 and T2.

	Pain	Neurological	Fatigue
Patients affected at T1 (*n*)	70	48	57
Patients affected at T2 (*n*)	41	30	47
Overall decrease in affected patients (%)	41.43	37.50	17.54
Paired *t*-test (*p* value)	<0.001	<0.01	>0.05
Cohen’s *d* (95% CI)	0.43 (0.32–0.53)	0.28 (0.18–0.38)	0.15 (0.04–0.25)
Cohen’s *d* interpretation	Medium	Medium	Small

**Table 5:** The number of patients prescribed with each type of combination antibiotic regimen.

Antibiotic Combination	Number of Patients, *n* (Percentage)
500 mg cefuroxime, 300 mg rifampicin and 300 mg lymecycline	76 (75.25)
500 mg cefuroxime, 300 mg rifampicin and 500 mg azithromycin	6 (5.94)
500 mg cefuroxime, 300 mg rifampicin and 500 mg clarithromycin	4 (3.96)
300 mg rifampicin, 300 mg lymecycline and 500 mg azithromycin	7 (6.93)
300 mg rifampicin, 300 mg lymecycline and 500 mg clarithromycin	1 (0.99)
1000 mg cefuroxime, 300 mg rifampicin and 300 mg lymecycline	1 (0.99)
500 mg cefuroxime and 300 mg rifampicin	4 (3.96)
300 mg rifampicin and 300 mg lymecycline	1 (0.99)
300 mg rifampicin and 500 mg azithromycin	1 (0.99)

## Data Availability

All of the relevant data are provided in the form of regular figures, tables, and Supplementary Materials file.

## Supplementary Materials

The following supporting information can be downloaded at: https://www.mdpi.com/article/10.3390/microorganisms11092152/s1, Table S1: Data for antibiotic tolerance, renal and liver function tests.
